# High and Low Protein∶ Carbohydrate Dietary Ratios during Gestation Alter Maternal-Fetal Cortisol Regulation in Pigs

**DOI:** 10.1371/journal.pone.0052748

**Published:** 2012-12-26

**Authors:** Ellen Kanitz, Winfried Otten, Margret Tuchscherer, Maria Gräbner, Klaus-Peter Brüssow, Charlotte Rehfeldt, Cornelia C. Metges

**Affiliations:** 1 Research Unit of Behavioural Physiology, Leibniz Institute for Farm Animal Biology, Dummerstorf, Germany; 2 Research Unit of Reproductive Biology, Leibniz Institute for Farm Animal Biology, Dummerstorf, Germany; 3 Research Unit of Muscle Biology and Growth, Leibniz Institute for Farm Animal Biology, Dummerstorf, Germany; 4 Research Unit of Nutritional Physiology “Oskar Kellner”, Leibniz Institute for Farm Animal Biology, Dummerstorf, Germany; University of Cordoba, Spain

## Abstract

Imbalanced maternal nutrition during gestation can cause alterations of the hypothalamic-pituitary-adrenal (HPA) system in offspring. The present study investigated the effects of maternal low- and high-protein diets during gestation in pigs on the maternal-fetal HPA regulation and expression of the glucocorticoid receptor (GR), mineralocorticoid receptor (MR), 11β-hydroxysteroid dehydrogenase 1 and 2 (11β-HSD1 and 11β-HSD2) and c-fos mRNAs in the placenta and fetal brain. Twenty-seven German Landrace sows were fed diets with high (HP, 30%), low (LP, 6.5%) or adequate (AP, 12.1%) protein levels made isoenergetic by varying the carbohydrate levels. On gestational day 94, fetuses were recovered under general anesthesia for the collection of blood, brain and placenta samples. The LP diet in sows increased salivary cortisol levels during gestation compared to the HP and AP sows and caused an increase of placental GR and c-fos mRNA expression. However, the diurnal rhythm of plasma cortisol was disturbed in both LP and HP sows. Total plasma cortisol concentrations in the umbilical cord vessels were elevated in fetuses from HP sows, whereas corticosteroid-binding globulin levels were decreased in LP fetuses. In the hypothalamus, LP fetuses displayed an enhanced mRNA expression of 11β-HSD1 and a reduced expression of c-fos. Additionally, the 11β-HSD2 mRNA expression was decreased in both LP and HP fetuses. The present results suggest that both low and high protein∶carbohydrate dietary ratios during gestation may alter the expression of genes encoding key determinants of glucocorticoid hormone action in the fetus with potential long-lasting consequences for stress adaptation and health.

## Introduction

Experimental and epidemiological studies have led to the hypothesis that exposure to adverse maternal environments during critical periods of fetal growth and development increases the risk of disease in the adult offspring [1]. One of the possible mechanisms by which prenatal influences can predispose to disease in later life is via changes in the hypothalamic-pituitary-adrenal (HPA) axis [2]. Up-regulation of the maternal HPA axis can result in elevated glucocorticoid concentrations in the offspring, contributing to the risk of hypertension, glucose intolerance and obesity [3]. This result is supported by the discovery of an association between reduced birth weight and increased plasma cortisol concentrations in adult human populations [4], [5].

Imbalanced maternal nutrition during gestation is an important environmental factor that may exert permanent effects on the developing fetus. Several studies have shown that undernutrition and/or protein deficiency during gestation may produce increased maternal glucocorticoid concentrations, which may restrict fetal growth and cause alterations in endocrine and metabolic systems [6], [7], [8]. On the other hand, an excess of maternal protein intake can also increase maternal glucocorticoid concentration with negative effects on birth weight, although studies have produced inconsistent results [9]–[11]. However, the precise mechanisms linking maternal malnutrition to reduced birth weight and metabolic disease in the offspring are still unknown.

Glucocorticoids, the end product of HPA axis activation, are presumed to represent a primary programming signal. Glucocorticoid hormone action within the cell is regulated by the expression of the glucocorticoid receptor (GR), mineralocorticoid receptor (MR) and both isoforms of 11β-hydroxysteroid dehydrogenase (11β-HSD1 and 11β-HSD2) at the level of gene expression [12]. 11β-HSD1 acts predominantly as an 11-oxoreductase, catalyzing the conversion of inactive cortisone to active cortisol, thereby amplifying activation of intracellular GR. Conversely, 11β-HSD2 behaves as a 11β-dehydrogenase, catalyzing the inactivation of cortisol to cortisone and thus, it protects the intrinsically non-selective MR and the selective GR from activation by cortisol [13]. Furthermore, 11β-HSD2 expression in fetal-placental tissues is assumed to protect the fetus from the deleterious effects of excess glucocorticoids [14], [15]. In the pig, the expression of placental 11β-HSD2 was demonstrated, and maternal cortisol was revealed to be converted into cortisone as it traverses the porcine placenta [16], [17]. With respect to the maternal dietary protein level, previous studies in pigs have shown that severe protein deficiency during gestation may decrease placental and fetal growth and affect body composition and postnatal growth [18], [19]. Recently, we have reported that inadequate maternal dietary protein and/or carbohydrate levels during gestation in primiparous sows cause reduced birth weight of the piglets, may affect cellular properties of skeletal muscle and subcutaneous adipose tissue in the offspring and may alter maternal macronutrient and fetal amino acid metabolism [20]–[24]. However, it is not clear whether inadequate dietary protein to carbohydrate ratios during gestation affect the maternal and developing fetal HPA axis in pigs and/or what the possible underlying mechanism may be. In view of the fact that the pig is increasingly recognized as a model for humans in biomedical research [25], [26], alterations in HPA axis regulation as a consequence of maternal protein diet are of particular interest with respect to human health and sustainable animal production. Therefore, the aim of the present study was to investigate the effects of low (6.5% protein) and high (30% protein) protein levels in the diet of sows throughout gestation on the maternal-fetal cortisol regulation and the expression of genes regulating the glucocorticoid response in the placenta and fetal brain. For this purpose, the salivary cortisol levels during gestation and the diurnal rhythmicity of cortisol in sows as well as the cortisol and corticosteroid-binding globulin (CBG) concentrations in the umbilical cord and fetuses were measured. Additionally, the mRNA expression levels of GR, MR, 11β-HSD1, 11β-HSD2 and c-fos were examined in the placenta and fetal hypothalamus and hippocampus. To characterize transplacental cortisol transfer, the enzyme activities of 11β-HSD1 and 11β-HSD2 were analyzed in the placenta.

## Materials and Methods

### Ethic statement

All of the procedures involving animal handling and treatment were in strict accordance with the German animal protection law and approved by the relevant authorities (Landesamt für Landwirtschaft, Lebensmittelsicherheit und Fischerei, Mecklenburg-Vorpommern, Germany; LVL M-V/TSD/7221.3-1.1-006/04; LALLF M-V/TSD/7221.3-1.2-050/06; LALLF M-V/TSD/7221.3-1.2-013/07). All surgery was performed under anesthesia (see below) and all efforts were made to minimize suffering.

### Animals and experimental procedures

A total of 27 primiparous German Landrace sows, bred and raised in the institute's pig breeding facility, were used. The experiment was conducted over five temporally successive replicates. Housing and breeding management were described in detail by Rehfeldt et al. [22]. One day prior to the first insemination, sows were randomly allocated to three dietary groups. The day of the second insemination was designated as gestational day (GD) 1. Starting on the day of the first insemination, sows were fed an isoenergetic corn-barley, soybean meal diet (∼13.7 MJ ME/kg) with target crude protein levels of 120 (adequate protein, AP; n = 9), 300 (high protein, HP; n = 9) or 60 g/kg (low protein, LP; n = 9) balanced by different carbohydrate concentrations throughout gestation. Consequently, in the AP, HP and LP diets, the protein∶carbohydrate ratios were 1∶5, 1∶1.3 and 1∶10.4, respectively. Diets were fed between 2.3 and 2.9 kg/d to achieve an average target energy intake of ∼34 MJ ME/d during gestation following the recommendations for primiparous sows [27]. The sows were fed twice daily, and water was provided ad libitum.

Salivary samples were collected daily between 08:00 and 10:00 h on GD −5 to −3, GD 22 to 24, GD 57 to 59 and GD 77 to 79. To collect serial blood samples for a circadian study, the animals were surgically fitted with indwelling jugular vein catheters on GD 84, as described by Metges et al. [20]. Beginning on GD 92 at 08:00 h, blood samples were collected every two hours until the next morning at 08:00 h. On GD 94, recovery of fetuses was performed by Caesarean section *lege artis* under general anesthesia with azaperone (0.05 ml/kg body weight Stresnil, Janssen-Cilag GmbH, Neuss, Germany) and ketamine (0.15 ml/kg body weight Ursotamin, Serumwerk Bernburg AG, Bernburg, Germany) administered via an implanted jugular vein catheter. Blood samples were taken from each sow immediately before surgery and during anesthesia via catheter as well as from the umbilical vein and artery of the first nine fetuses taken after the initiation of anesthesia by puncture. After recovery, placental samples from the fetal implantation sites (chorionic plates) were rapidly dissected and prepared for RNA and enzyme activity analyses. Fetal blood was obtained by puncture of the vena cava cranialis, and immediately thereafter, fetuses were euthanized by an injection of T61® (200 mg embutramide, 50 mg mebezonium iodide and 5 mg tetracaine hydrochloride, Intervet, Unterschleissheim, Germany). The body weight and length (crown to rump) of fetuses were recorded, and the brains were then quickly removed, weighed and placed on sterile, ice-cold Petri-dishes. The hypothalamus, including the paraventricular nucleus (PVN), and the hippocampus were dissected for RNA analyses. The stereotaxic atlas of the pig brain served as a reference [28]. After the recovery of the last fetus, the sows were euthanized by intravenous injection of T61® (16 mg embutramide, 2.4 mg mebezonium iodide and 0.4 mg tetracaine hydrochloride per kg body weight). All experimental procedures were performed on the animals between 07:30 and 11:00 h. All fetuses recovered from each sow were used for the evaluation of litter size, fetal body weight, crown-rump length and brain weight.

### Saliva and blood sampling and tissue preparation

Saliva samples were collected before feeding by allowing the sows to chew on veterinary cotton buds until these were thoroughly moistened (approximately 30–60 s per sample). The buds were placed in tubes and centrifuged at 2,500 *g* for 15 min at 4°C. Saliva samples were stored at −20°C until analysis. After thawing, the samples were spun at 2,500 *g* for 5 min, resulting in a clear supernatant with low viscosity.

Blood samples were collected in ice-cold polypropylene tubes containing EDTA solution, placed on ice and subsequently centrifuged at 2,000 *g* for 15 min at 4°C to separate the plasma portion. Plasma was then stored at −20°C until it was analyzed for their cortisol and corticosteroid-binding globulin (CBG) concentrations.

For mRNA expression analysis, the hypothalamus, hippocampus and placental samples were incubated overnight in RNAlater (RNA Stabilization Reagent, Qiagen, Hilden, Germany) at 2–4°C to protect RNA integrity and then transferred to −80°C for storage. For analysis of 11β-HSD activity, placental tissue was frozen in liquid nitrogen and stored at −80°C.

### Cortisol analyses

Cortisol concentrations in 25 µl saliva samples were measured in duplicate by an enzyme immunoassay (ACTIVE Cortisol EIA DSL-10-67100, Diagnostic Systems Laboratories Inc., Sinsheim, Germany) according to the instructions of the manufacturer. The cross-reactivity of the cortisol antiserum was measured against various compounds and was 58.3% for prednisolone, 10.9% for prednisone, 7.0% for cortisone, 5.7% for 11-deoxycortisol, 1.9% for 21-deoxycortisol, 0.9% for 17α-hydroxycortisol and dexamethasone and 0.4% for triamcinolone according to the manufacturer. The lowest level of cortisol that can be detected by this assay in porcine saliva was 0.35 ng/ml, and the intra- and inter-assay coefficients of variation (CVs) were 2.0% and 8.7%, respectively.

Plasma cortisol concentrations were analyzed in duplicate using a commercially available ^125^I-RIA kit (ACTIVE Cortisol RIA DSL-2100, Diagnostic Systems Laboratories Inc., Sinsheim, Germany) according to the manufacturer's guidelines. Cross-reactivities of the antibody used to prednisolone and corticosterone were 33.3% and 9.3%, respectively, and to any further competing plasma steroids lower than 5%. The assay was validated for use with porcine plasma. The test sensitivity was 8.1 nmol/l, and the intra- and inter-assay CVs were 8.2% and 9.8%.

### Corticosteroid-binding globulin (CBG)

Plasma samples were examined for CBG using a modified binding assay previously described by Kanitz et al. [29]. Briefly, after removing endogenous steroids from the plasma by dextran-coated charcoal treatment, 25 µl of plasma was incubated with 0.78 nM unlabeled cortisol (Hydrocortisone, Merck, Darmstadt, Germany) and 25 pM ^3^H-cortisol (specific radioactivity 68 Ci/mmol, Amersham Pharmacia Biotech, Freiburg, Germany). Non-specific binding (e.g. albumin binding) was determined in parallel using a 100-fold excess of cold cortisol. The separation of bound and free ^3^H-cortisol was performed by precipitation with dextran-coated charcoal at 4°C and subsequent centrifugation at 1,000 *g* for 10 min. Aliquots of supernatant fraction were taken for scintillation counting. CBG binding was calculated as the difference between the total binding and the non-specific binding of cortisol. The intra- and inter-assay CVs were 7.8% and 9.1%.

The Free Cortisol Index (FCI), a surrogate measure of plasma free cortisol, was calculated using the following formula: FCI = [cortisol]/[CBG].

### RNA extraction and quantification of transcripts

Total RNA from individual hypothalamus, hippocampus and placenta samples was extracted using the Invisorb® Spin Tissue RNA Mini Kit (Invitek, Berlin, Germany) according to the manufacturer's protocol. The concentration of RNA was determined by the absorbance at 260 nm. The RNA purity and integrity were assessed by the 260/280 nm ratio and electrophoresis using 2% agarose in TBE buffer (89 mM Tris, 89 mM boric acid, 1 mM EDTA, pH 8.0) and the SYBR gold stain (MoBiTec, Göttingen, Germany) at a final concentration recommended by the manufacturer.

The absolute quantification of mRNA using the real-time reverse transcriptase chain reaction (RT-PCR) was performed as previously described by Löhrke et al. [30]. Fragments of mRNA encoding GR, MR, 11β-HSD1 and 11β-HSD2 were obtained from reverse transcription (RT) performed by an iScript cDNA synthesis kit (BIO-RAD, München, Germany) with 100 ng of total RNA following the guidelines of the manufacturer. The resulting cDNA was amplified by real-time PCR (iCycler, BIO-RAD, München, Germany) using an iQ SYBR green supermix (BIO-RAD, München, Germany). One microliter of RT reaction was added to 10 µl of PCR mix primed with gene-specific oligonucleotides (TIB MOLBIOL, Berlin, Germany). PCR was carried out using a hot start (3 min, 94°C; 30 s, 60°C; 45 s, 70°C) followed by 45 cycles (10 s, 94°C; 30 s, 60°C; 45 s, 70°C with 5 s added in each cycle) and with a final cycle of 10 s, 94°C; 30 s, 60°C; 7 min, 70°C for denaturation, annealing and elongation, respectively. The primers were designed to span a corresponding intron and to anneal between 60°C and 70°C based on the published cDNA and gene sequences (for GR, accession no. AY779185; for MR, accession no. M36074; for 11β-HSD1, accession no. NM_008288; for 11β-HSD2, accession no. AF414125; for c-fos, accession no. AJ132510). The following primer sequences were used: GR (forward, 5′-GTT CCA GAG AAC CCC AAG AGT TCA-3′; reverse, 5′-TCA AAG GTG CTT TGG TCT GTG GTA-3′), MR (forward, 5′-GTC TTC TTC AAA AGA GCC GTG GAA-3′; reverse, 5′-CTC CTC GTG GAG GCC TTT TAA CTT-3′), 11β-HSD1 (forward, 5′-GGT CAA CTT CCT CAG CTA CGT GGT-3′; reverse, 5′-AGG ACA CAG AGA GTG ATG GAC ACG-3′), 11β-HSD2 (forward, 5′-TGG TAC CCT TGA GAT GAC CAA-3′; reverse, 5′-CAC TGG TCC ACG TTT TTC ACT-3′), c-fos (forward, 5′-GGG ACA GTC TCT CCT ACT ACC ACT-3′; reverse, 5′-GGT GAG GGG CTC TGG TCT-3′).

The specificity of the products was assessed using a melting point analysis starting at 60°C and elevating the temperature to 90°C (1°C per 10 s), as well as by agarose gel electrophoresis in comparison with oligonucleotide molecular mass ladders to confirm that the calculated molecular mass of the product corresponds to the produced oligonucleotide. The oligonucleotide structure was checked by sequencing in some experiments. The mRNA abundance was calculated using the known concentration of standard oligonucleotides and amplification efficiency displayed by the iCycler and expressed as pg per µg of total RNA.

### 11β-hydroxysteroid dehydrogenase (11β-HSD) activity

Placental 11β-HSD activity was measured by a modified radiometric conversion assay as previously described [16], [31]. Tissue was homogenized in Krebs-Ringer bicarbonate buffer (118 mM NaCl, 3.8 mM KCl, 1.19 mM KH_2_PO_4_, 2.54 mM CaCl_2_ × 2H_2_O, 1.19 mM MgSO_4_ × 7H_2_O, 25 mM NaHCO_3_, and 0.2% glucose; pH 7.4), and the homogenate was centrifuged at 4°C and 1,000 g for 10 min. The protein concentration was measured by the Lowry method [32]. For 11β-HSD dehydrogenase activity, an aliquot of homogenate (0.5 mg protein/ml) was incubated in 0.5 ml Krebs-Ringer buffer (+0.2% bovine serum albumin) containing 12 nM [^3^H]-cortisol ([1,2,6,7-^3^H]-cortisol; specific activity 70 Ci/mmol; Amersham Pharmacia Biotech Europe GmbH, Germany), 1 µM unlabeled cortisol and 400 µM NADP^+^ or NAD^+^ (Sigma Aldrich GmbH, Germany) for 1 h at 37°C in a shaking water bath. While the enzyme activity measured in the presence of NADP^+^ is attributed to 11β-HSD1, the net oxidation of cortisol to cortisone in the presence of NAD^+^ will be the sum of the activities of both 11β-HSD1 and 11β-HSD2 isoenzymes. The 11β-HSD1 reductase activity was assessed by measuring the rate of conversion of [^3^H]-cortisone to [^3^H]-cortisol. It was determined by incubating homogenate (0.5 mg protein/ml) with 1 µM cortisone (Sigma Aldrich GmbH, Germany) and [^3^H]-cortisone ([1,2(n)-^3^H]-cortisone; specific activity 45 Ci/mmol; Amersham Pharmacia Biotech Europe, Germany) as a tracer in the presence of 400 µM NADPH at 37°C for 1 h. Following all incubations, the transfer of the reaction mixtures into 4 ml ice-cold ethyl acetate stopped the reaction and extracted the steroids into organic phase, which was dried under nitrogen. Steroids were separated by thin-layer chromatography on silica gel 60 F_254_ plates (Merck KGaA, Germany) using 92∶8 chloroform, methanol mobile phase. The bands were visualized under UV light, and the activity was quantified using a liquid scintillation counter (Tri-Carb 2900TR, Perkin Elmer LAS GmbH, Germany). All samples were assayed in duplicate and specific conversions of [^3^H]-cortisol or [^3^H]-cortisone into labeled cortisone or cortisol, respectively, were calculated by subtracting non-specific conversion in the presence of a heat-denatured placental preparation from total conversion. 11β-HSD activities were expressed as nmol cortisone or cortisol formed/mg protein per 1 h.

### Statistical analyses

The experiment was conducted over five replicates with all three dietary treatments present in each replicate. For each of the AP, HP and LP dietary groups, a total of 9 sows were included in the statistical analysis. The data were evaluated using SAS/STAT ® 9.2 [33]. Repeated measures ANOVA (PROC MIXED) was used to evaluate the effects of diet on salivary cortisol and the circadian rhythm of plasma cortisol concentrations. The model included the fixed factor diet, replicate, the repeated factor time, the diet × replicate and diet × time interactions and the random factor sow within diet and replicate. Areas under the curve (AUCs) were calculated for plasma cortisol concentrations measured over a period of 24 h using the trapezoidal method [34]. Fetal data (hormones, enzyme activity and mRNA expression) were analyzed with the PROC MIXED model using the fixed factors diet, replicate, sex, the interactions diet × sex, diet × replicate and the random factor sow within diet and replicate. The number of fetuses per litter was analyzed with the mixed model comprising the fixed factors diet, replicate and the interaction diet × replicate. The model for body weight, crown-rump length and weight of brain related to body weight included the fixed factors diet, replicate, sex, litter size group (litters <13 and ≥13 piglets according to 13 as the median of litter size), the interactions diet × sex, diet × replicate, diet × litter size group and the random factor sow within diet, replicate and litter size group.

Comparisons between different levels within factors were made using the Tukey-Kramer test. The results are reported as least square means (LS means) ± standard error (SE).

Spearman's rank correlation coefficients were estimated between maternal diurnal cortisol concentrations (area under curve, AUC) and endocrine measures in the placenta, umbilical vessels and fetuses and tested (*vs*. 0) in the AP, HP and LP dietary groups using the CORR procedure.

The significance level was set at *P*≤0.05.

## Results

### Effects of diet on cortisol concentrations in sows

**Figure 1:**
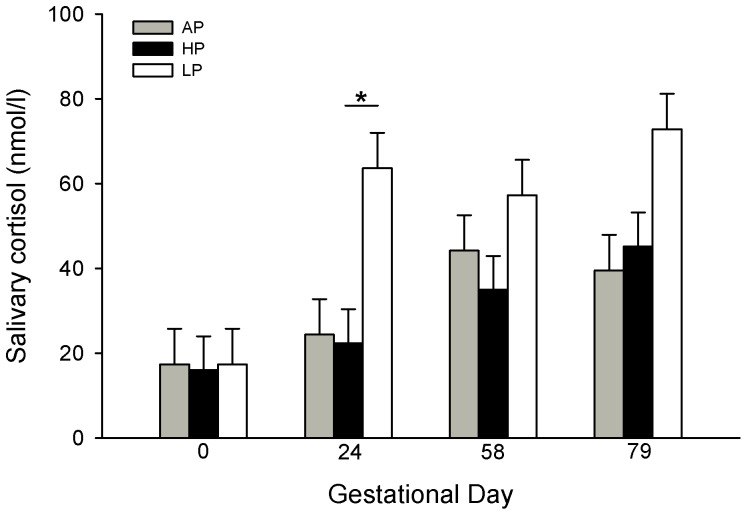
Salivary cortisol concentrations of primiparous sows throughout gestation. Sows were fed diets with adequate (AP, 12.1%; n = 9), high (HP, 30%; n = 9) or low (LP, 6.5%; n = 9) protein concentrations and salivary cortisol was measured prior to gestation (GD 0) and on GD 24, 58 and 79. Values are LS means + SE. Significant differences between diet groups are indicated by asterisks (*P<0.05). Salivary cortisol concentrations were higher in the LP sows compared to the HP and AP sows.

The salivary cortisol concentrations of sows during gestation were influenced by diet (*P*<0.01), time (*P*<0.001) and the diet × time interaction (*P*<0.05). Pairwise comparison of the diet groups revealed higher salivary cortisol levels in LP sows compared to AP (*P*<0.05) and HP (*P*<0.05) sows during gestation. Multiple comparisons within the time points by the Tukey-Kramer procedure provided a significantly increased salivary cortisol concentration in LP sows compared to HP sows on GD 24 (Figure 1).

**Figure 2:**
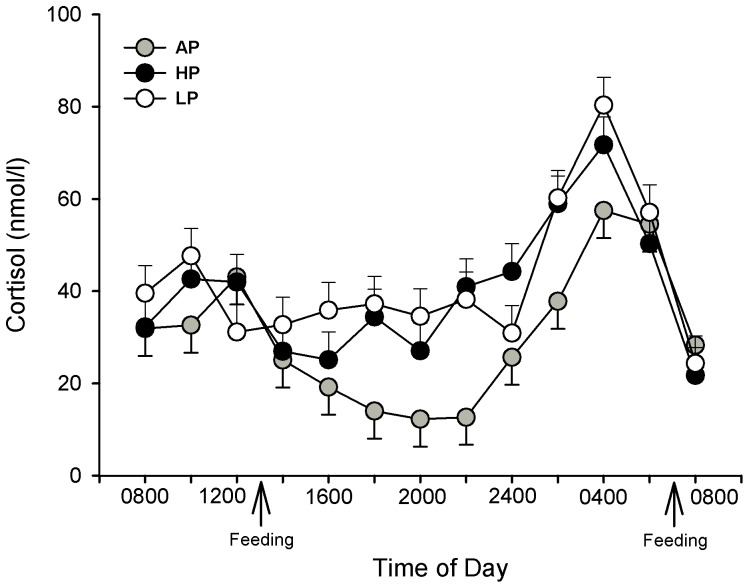
Diurnal plasma cortisol concentrations in primiparous sows on GD 92. Sows were fed diets with adequate (AP, 12.1%; n = 9), high (HP, 30%; n = 9) or low (LP, 6.5%; n = 9) protein concentrations throughout gestation. Cortisol concentrations were measured over a 24-h period beginning on GD 92. Values are LS means ± SE. The calculated AUC of the HP and LP sows displayed higher cortisol concentrations over 24 h compared to the AP sows (*P*<0.05).

Statistical analyses revealed a significant effect of diet (*P*<0.001) and time (*P*<0.001) on the circadian plasma cortisol concentrations of sows on GD 91. The dynamic in the plasma profile of cortisol is shown in Figure 2. A normal periodicity was observed in the AP sows, with high levels in the early morning and a nadir at 2000. There was a 4.7-fold increase (12.3 nmol/l vs. 57.5 nmol/l; *P*<0.001) from the minimum (2000) to maximum (0400) cortisol concentrations in these sows. In contrast, the HP and LP sows displayed unchanged concentrations during a period from 0800 to 2000 and a 2.6- and 2.3-fold increase of cortisol to maximum levels at 0400, respectively. When plasma cortisol concentrations were calculated as the total area under the curve (AUC), statistical analyses revealed that the AUC was greater in the LP (1035.6±56.5 nmol/l h) and HP (974.7±59.3 nmol/l h) sows compared to the AP (729.1±56.5 nmol/l h) sows (LP vs. AP: *P*<0.01; HP vs. AP: *P*<0.05).

Immediately before fetus recovery, maternal plasma cortisol concentrations were not different between the diets (*P*>0.81). The anesthesia caused an approximately 3.5-fold increase of cortisol concentration in all three diet groups (*P*<0.01; data not shown), but there was no effect of diet on cortisol concentration during Caesarean section (*P*>0.36). Furthermore, the cortisol concentration did not significantly change with the recovery of the first up to the ninth fetus (*P*>0.29).

### Effects of maternal diet on placenta

**Figure 3:**
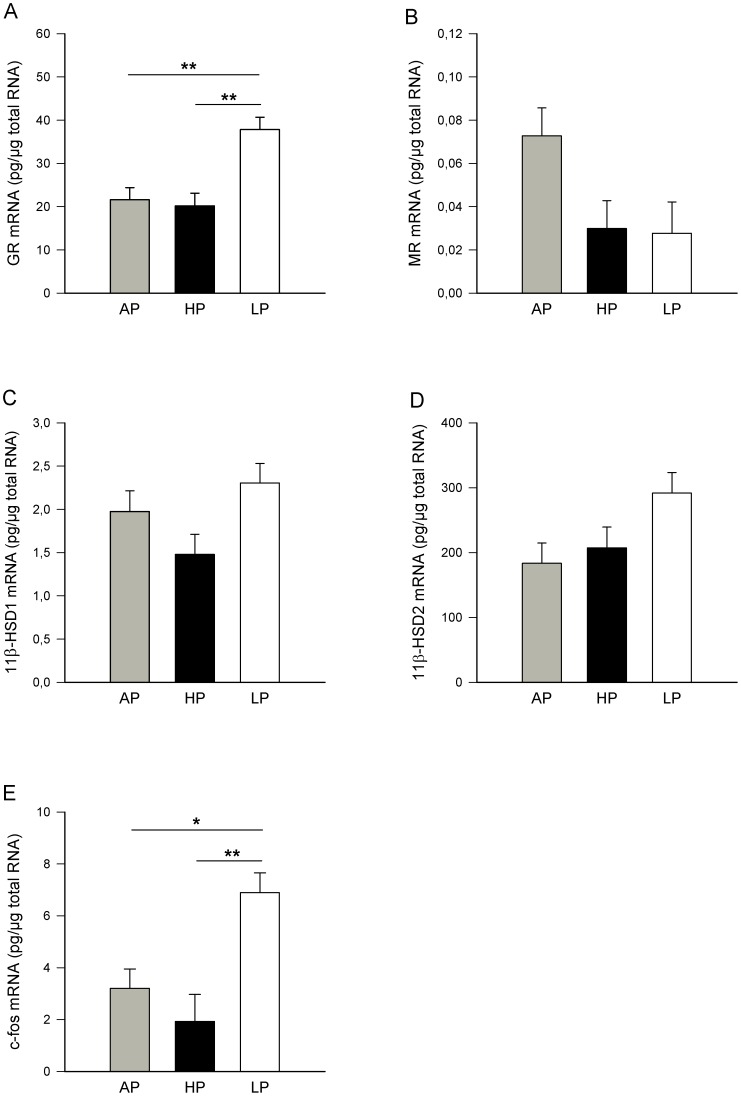
mRNA expression in placentas on GD 94. Sows were fed diets with adequate (AP, 12.1%), high (HP, 30%) and low (LP, 6.5%) protein concentrations throughout gestation and the mRNA expression of the glucocorticoid receptor (GR), mineralocorticoid receptor (MR), 11β-hydroxysteroid dehydrogenase type 1 (11β-HSD1), 11β-hydroxysteroid dehydrogenase type 2 (11β-HSD2) and c-fos in placentas from the fetal implantation site was measured on GD 94. Values are LS means + SE. Significant differences between diet groups are indicated by asterisks (*P<0.05; **P<0.01). A: GR mRNA expression was higher in placentas from the LP fetuses (n = 46) compared to the HP (n = 54) and AP (n = 48) fetuses. B: MR mRNA expression in placentas from the AP (n = 43), HP (n = 48) and LP (n = 35) fetuses. C: 11β-HSD1 mRNA expression in placentas from the AP (n = 36), HP (n = 50) and LP (n = 44) fetuses. D: 11β-HSD2 mRNA expression in placentas from the AP (n = 48), HP (n = 54) and LP (n = 43) fetuses. E: c-fos mRNA expression was higher in placentas from the LP (n = 46) fetuses compared to the HP (n = 50) and AP (n = 50) fetuses.

ANOVA revealed significant effects of diet on placental GR mRNA (*P*<0.01) and c-fos mRNA (*P*<0.01) expression, whereas no significant effects were found for placental MR mRNA (P = 0.06), 11β-HSD1mRNA (P = 0.07) and 11β-HSD2 mRNA (P = 0.07) expression. Tukey-Kramer tests showed that the LP diet caused significantly higher GR mRNA expression (Figure 3A) compared to AP (*P*<0.01) and HP (*P*<0.01) diets and higher c-fos mRNA expression compared to AP (*P*<0.05) and HP (*P*<0.01) sows (Figure 3E).

**Figure 4:**
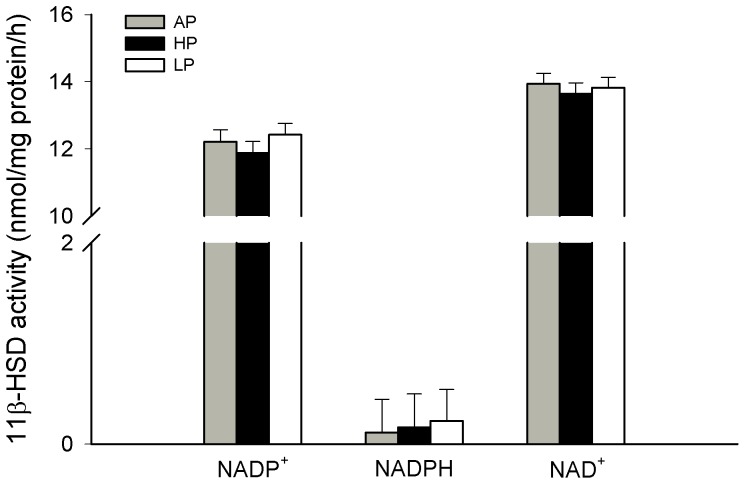
Enzymatic activities of 11β-hydroxysteroid dehydrogenase in placentas on GD 94. Sows were fed diets with adequate (AP, 12.1%), high (HP, 30%) and low (LP, 6.5%) protein concentrations throughout gestation and the enzymatic activities of 11β-hydroxysteroid dehydrogenase (11β-HSD) in placentas from the fetal implantation site were measured on GD 94. Values are LS means + SE. The maternal diets had no effect on the NADP^+^-dependent (NADP^+^; AP, n = 13; HP, n = 14; LP, n = 16) or NAD^+^-dependent 11β-HSD dehydrogenase activity (NAD+; AP, n = 17; HP, n = 16; LP, n = 17) as well as on the NADPH-dependent 11β-HSD reductase activity (NADP; AP, n = 15; HP, n = 16; LP, n = 17).

There was no effect of diet during gestation on enzyme activity of 11β-HSD in the placenta (*P*>0.66). The highest enzyme activity was measured for NAD^+^-dependent 11β-HSD dehydrogenase activity compared to the NADP^+^-dependent activity (*P*<0.001), which reflects 11β-HSD1 activity, and NADPH-dependent 11β-HSD1 reductase activity (*P*<0.001; Figure 4).

To demonstrate the relationships between the placental mRNA expression of GR, MR, 11β-HSD1, 11β-HSD2 and the activity of 11β-HSD and circadian maternal cortisol concentrations (AUC) before fetus recovery, Spearman's rank correlation coefficients were determined for the three dietary groups (AP: n = 36–50; HP: n = 41–50; LP: n = 32–46). There was a negative relationship between placental GR mRNA and diurnal cortisol concentration in HP sows (r_s_ = −0.2907; *P*<0.05) and between MR mRNA and maternal cortisol in AP sows (r_s_ = −0.5636; *P*<0.001). No further significant correlations were found for placental variables and maternal diurnal cortisol.

### Effects of maternal diet on fetuses

There was a main effect of maternal dietary protein:carbohydrate ratios on body weight (*P*<0.01) and the relative brain weight (*P*<0.05) of fetuses. As shown in Table 1, the body weights of HP (*P*<0.01) and LP (*P* = 0.05) fetuses were lower compared to AP fetuses, and the brain weight related to body weight was higher in the fetuses from mothers fed the HP diet compared to the AP diet (*P*<0.05). The number of fetuses per litter and the crown-rump length were not significantly affected by maternal diet (*P*>0.20).

**Table 1:** Litter size, body weight and length and brain weight of fetuses recovered on GD 94 from sows fed isoenergetic diets with adequate (AP, 12.1%; n = 9), high (HP, 30%; n = 9) and low (LP, 6.5%; n = 9) protein concentrations throughout gestation.

	AP	HP	LP
Number of fetuses/litter	13.2±1.2	12.4±1.3	12.3±1.2
Fetus body weight (g)	703.5±16.4^a,c^	615.1±15.9^b,d^	639.8±16.1^b^
Crown-rump length (cm)	25.4±0.3	24.6±0.3	25.0±0.3
Brain (g/kg body weight)	27.0±0.9^a^	31.7±0.9^b^	28.6±0.9

Values are LS means ± SE (AP, n = 114; HP, n = 107; LP, n = 108). Significant differences between diet groups are indicated by different superscripts within a row (^a,b^ *P*≤0.05; ^c,d^ *P*<0.01). The body weight was significantly lower in HP and LP fetuses, and the brain weight related to body weight was significantly higher in fetuses of HP sows compared to AP fetuses.

**Figure 5:**
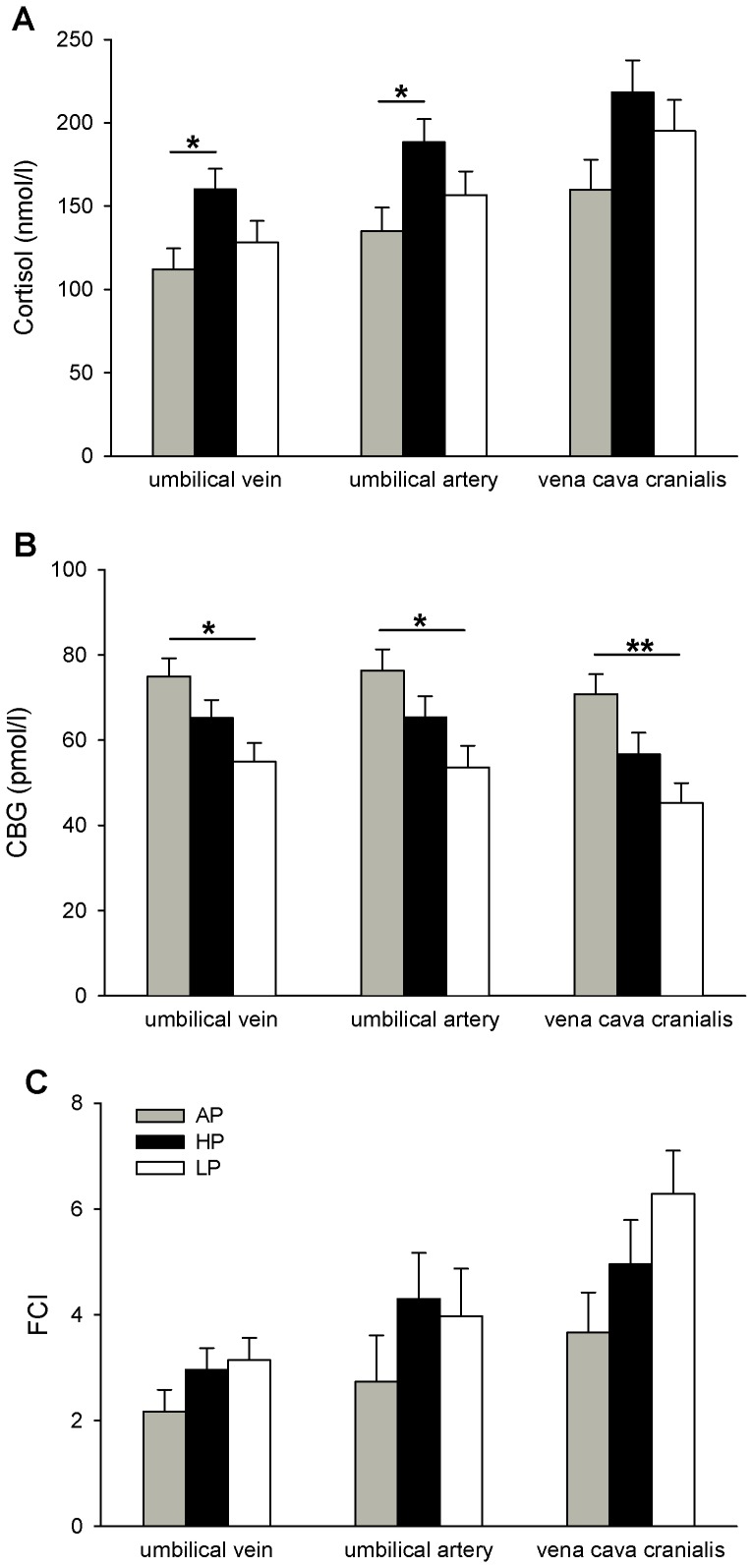
Cortisol, corticosteroid-binding globulin and free cortisol index in cord blood and fetuses on GD 94. Sows were fed diets with adequate (AP, 12.1%), high (HP, 30%) and low (LP, 6.5%) protein concentrations throughout gestation and the cortisol and corticosteroid-binding globulin (CBG) concentrations and free cortisol index (FCI) in the endogenous (vena cava cranialis) and umbilical blood vessels of fetuses were measured on GD 94. Values are LS means + SE (AP, n = 80; HP, n = 81; LP, n = 80). Significant differences between diet groups are indicated by asterisks (*P<0.05; **P<0.01). A: cortisol concentrations were higher in the umbilical veins and arteries from HP fetuses compared to controls. B: CBG concentrations were reduced in the endogenous and umbilical blood vessels of LP fetuses compared to controls. C: calculated FCI in the endogenous and umbilical blood vessels of AP, HP and LP fetuses.

**Figure 6:**
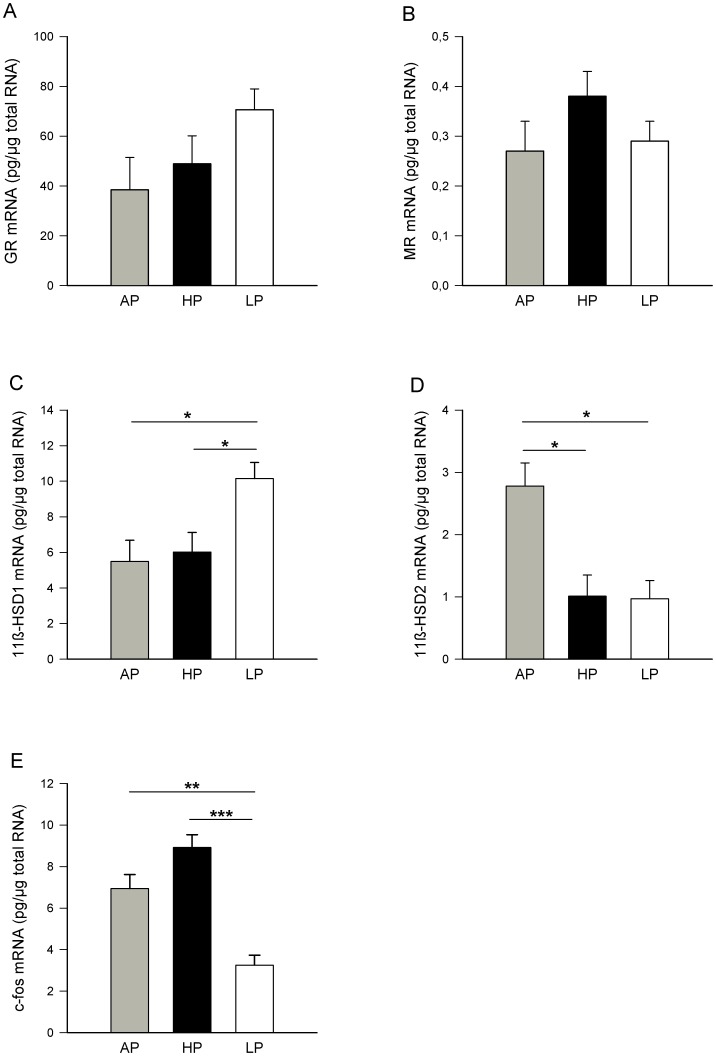
mRNA expression in fetal hypothalami on GD 94. Sows were fed diets with adequate (AP, 12.1%), high (HP, 30%) and low (LP, 6.5%) protein concentrations throughout gestation and the mRNA expression of glucocorticoid receptor (GR), mineralocorticoid receptor (MR), 11β-hydroxysteroid dehydrogenase type 1 (11β-HSD1), 11β-hydroxysteroid dehydrogenase type 2 (11β-HSD2) and c-fos in fetal hypothalami was measured on GD 94. Values are LS means + SE (AP, n = 15; HP, n = 17; LP, n = 18). Significant differences between diet groups are indicated by asterisks (*P<0.05; **P<0.01; ***P<0.001). A: GR mRNA expression in the hypothalamus from AP, HP and LP fetuses. B: MR mRNA expression in hypothalami from AP, HP, LP fetuses. C: 11β-HSD1 mRNA expression in the hypothalamus was increased in LP fetuses compared to HP and AP fetuses. D: 11β-HSD2 mRNA expression in the hypothalamus was reduced in both HP and LP fetuses compared to controls. E: c-fos mRNA expression was reduced in LP fetuses compared to HP and AP fetuses.

ANOVA indicated effects of the maternal diet on plasma cortisol concentrations in venous (*P*<0.05) and arterial (*P*<0.05) fetal cord blood, with Tukey-Kramer analysis revealing that HP fetuses displayed higher cortisol concentrations in the umbilical vein (*P*<0.05) and artery (*P*<0.05) compared to AP fetuses (Figure 5A). Further, the CBG concentrations in umbilical vessels and vena cava cranialis were affected by maternal diet (venous and arterial: *P*<0.05: vena cava cranialis: *P*<0.01). As shown in Figure 5B, the CBG concentrations were significantly reduced in all three blood vessels of the LP fetuses in comparison with the AP fetuses (umbilical vein and artery: *P*<0.05; vena cava cranialis: *P*<0.01). No effects of the maternal diet were found on the calculated FCI. The FCI tended to be higher in blood samples from the vena cava cranialis of the LP fetuses (*P* = 0.09; Figure 5C) compared to the AP fetuses. Correlation analysis indicated a positive relationship between diurnal cortisol concentration in LP sows and fetal endogenous total plasma cortisol (r_s_ = +0.3980; *P*<0.001) and FCI (r_s_ = +0.4823; *P*<0.0001). Maternal cortisol and fetal CBG concentrations were negatively correlated in sows of the LP (r_s_ = −0.4024; *P*<0.001) and HP (r_s_ = −0.2881; *P*<0.05) groups. We could not detect any relationships between diurnal maternal cortisol and fetal blood concentration variables in AP sows. In the fetal brain, there was an effect of diet on hypothalamic 11β-HSD1 (*P*<0.05), 11β-HSD2 (*P*<0.05) and c-fos (*P*<0.001) mRNA expression, whereas GR and MR transcripts were not significantly affected by the diet (*P*>0.14; Figure 6A, B). The LP fetuses displayed higher 11β-HSD1 mRNA levels than the AP and HP (*P*<0.05) fetuses (Figure 6C). On the other hand, the 11β-HSD2 mRNA expression was reduced in the hypothalamus from the LP and HP fetuses compared to the AP fetuses (*P*<0.05). Furthermore, the LP fetuses had lower c-fos mRNA expression compared to fetuses from mothers fed the AP (*P*<0.01) and HP (*P*<0.001) diets (Figure 6E). In the hippocampus, ANOVA revealed no significant effects of the maternal diet on GR, MR, 11β-HSD1, 11β-HSD2 or c-fos mRNA expression (*P*>0.11).

Correlation analyses indicated positive relationships between maternal diurnal cortisol concentrations and the GR (r_s_ = +0.8505; *P*<0.0001), MR (r_s_ = +0.7453; *P*<0.001) and 11β-HSD1 (r_s_ = +0.6782; *P*<0.01) mRNA expression in the fetal hypothalamus only in AP sows. However, a negative correlation was found between maternal cortisol concentration and 11β-HSD1 mRNA (r_s_ = −0.4247; *P*<0.05) in the hippocampus of LP fetuses. No further relationships were detected between maternal cortisol concentration and fetal brain variables.

The factor sex and the interaction diet × sex had no significant effects on any of the traits investigated (*P*>0.35).

## Discussion

The major novel findings in this porcine model demonstrate for the first time that both maternal diets with low protein to high carbohydrate ratios and high protein to low carbohydrate ratios throughout gestation generate changes in hormone concentrations and in the expression levels of genes encoding key receptors and enzymes that contribute to the regulation of the fetal cortisol levels and action. These alterations included increased maternal cortisol levels, dysregulated diurnal cortisol rhythm and heterogeneous diet-dependent effects on GR, 11β-HSD1, 11β-HSD2, c-fos mRNA expression in the placenta and fetal hypothalamus and on fetal plasma cortisol and CBG concentrations.

Our results showed a significant effect of gestation stage on maternal salivary cortisol with increasing concentrations in all three diet groups. A previous study in pigs also indicated an increase of maternal and fetal plasma cortisol until parturition [35], [36]. However, the LP diet caused higher salivary cortisol concentrations compared to AP and HP sows; this result reflects a higher concentration of unbound biologically active cortisol in LP sows. Additionally, we found a disruption of the normal daily rhythm in the circulating plasma cortisol concentration in LP sows at the end of gestation, with high levels maintained throughout the day. These results confirm previous studies on maternal undernutrition in human and animals. It is known that protein-caloric malnutrition, which is the most common condition found in humans [37], disturbs the energy balance of the organism. One of the crucial endogenous responses to preserve this balance is the activation of the HPA axis. Indeed, it is well established that food restriction and starvation may influence both the activity and rhythmicity of this axis [38]–[40]. Interestingly, in the present study, the HP diet also caused a disruption of the circadian rhythm in the plasma cortisol and a higher daily response (AUC), but had no effect on salivary cortisol as was the case in LP sows. The differences between fasted salivary and circadian plasma cortisol in HP sows may result from the varying sampling times and different metabolic state at these moments. We found that the circadian pattern of plasma cortisol is inversely proportional to the circadian pattern of glucose, showing a glucose deficit in HP and LP sows [20]. This glucose deficit may be counteracted in both diets by increased cortisol secretion to promote glycogenolysis and gluconeogenesis. In addition to this induced cortisol increase in both diets, we assume that during fasting conditions at the time of saliva sampling, protein deficiency in LP sows may further increase cortisol secretion in order to promote protein degradation.

It is known that maternal protein deficiency and also protein excess during gestation may cause intrauterine growth retardation and low birth weight in the offspring [7], [9], [10]. With respect to the present study, we found also a reduction of body weight in HP and LP fetuses on GD 94, which also supports previous observations in newborn piglets [22]. Additionally, an effect of maternal diet on the relative brain weight was found, with higher brain weights in the fetuses of HP sows. To date, the effects on fetal brain development were only described for maternal undernutrition [41]. It was demonstrated that the availability of nutrients plays a critical role in the maturation of the brain and in the development of optimal cognitive capacity [42]. As diet-derived amino acids are essential for the synthesis of structural proteins, enzymes and neuropeptides with influences on anatomy, chemistry and neurophysiology, the protein excess in the present study may be responsible for the higher brain weight in HP fetuses.

During gestation, glucocorticoids are important for fetal growth, tissue development and the maturation of various organs to prepare the fetus for extra-uterine existence. However, the development of the HPA axis in fetuses is protected from an excess supply of maternal glucocorticoids by placental 11β-HSD2, which catalyzes the rapid metabolism of cortisol to the inert 11-keto product cortisone [43]. This placental enzymatic barrier is not complete, as a minor proportion of maternal glucocorticoid crosses intact to the fetus; thus, maternal stress elevates fetal glucocorticoid levels [44]. It was also demonstrated for pigs that maternal cortisol provided 50% of the fetal cortisone during mid- and late gestation [45]. In the present study, the analysis of the 11β-HSD enzyme activity in placental tissues demonstrated the presence of both the 11β-HSD2 and 11β-HSD1 isoforms with a remarkably higher dehydrogenase activity compared to the reductase activity on GD 94. This distribution of NADP^+^- and NAD^+^-dependent activities was concordant with similarly high mRNA expression of 11β-HSD2 and low expression of 11β-HSD1 in the placenta. For pigs, Klemcke [46] also described placental 11β-HSD reductase activity in tissue fragment cultures on day 75 that was substantially less than the dehydrogenase activity, and a predominance of 11β-HSD dehydrogenase activity was also found in the placentae of other species [47], [48]. In regards to diet effects during gestation, studies in rats and sheep have shown that a maternal low protein diet or nutrient restriction reduces placental 11ß-HSD2 expression and programs increased glucocorticoid sensitivity at a tissue-specific level in the offspring [7], [49], [50]. In our study, we could not find any alterations in the placental 11β-HSD activity or mRNA expression of sows fed the LP or HP diets. This may be caused by the specific epitheliochorial nature of the porcine placenta in contrast to other species and/or the moderate diet-induced maternal cortisol increase. Indeed, LP sows displayed a higher expression of placental GR mRNA and c-fos mRNA. This increased expression indicates an enhanced cellular activation and local glucocorticoid hormone action within the placenta of LP sows, probably caused by higher free cortisol levels in these animals. An increase of placental GR mRNA abundance was also found after nutrient restriction in sheep, which may result in hypersensitivity of the offspring to glucocorticoids [51].

HPA activity is regulated by a negative feedback process in which circulating glucocorticoids act at various target sites in the brain and pituitary gland. CBG influences the bioavailability as well as the metabolic clearance rate of cortisol within the circulation [52]. The amount of cortisol that is taken up by target tissue is approximated by the non-CBG bound (free + albumin) fraction of total circulating steroid [53]. Thus, differences in circulating CBG levels determine the magnitude of the glucocorticoid negative feedback signal [54]. The present study demonstrated elevated cortisol concentrations in the umbilical vessels of HP fetuses compared to controls, whereas the CBG concentrations were reduced in umbilical and endogenous vessels from LP fetuses. However, the calculated free cortisol index (FCI) tended to reflect elevated endogenous concentrations of biologically active cortisol in LP fetuses. A positive correlation between the maternal diurnal cortisol and fetal cortisol as well as FCI was only found in LP fetuses, whereas a negative correlation was revealed between diurnal maternal cortisol and CBG in both HP and LP fetuses. These results indicate that the elevated maternal cortisol concentrations of both diets may affect fetal cortisol metabolism; however, the bioavailability of cortisol is adjusted by CBG and the effect of HP diet on fetal CBG concentrations seems to be weaker than in LP fetuses. It has not been directly demonstrated whether changes in plasma CBG result from changes in production, metabolic degradation and/or transfer to target tissues. However, Heo et al. [55] found in fetal pigs a positive correlation between plasma CBG concentrations and hepatic CBG mRNA. Therefore, the decrease of CBG concentration in LP fetuses in the present study may result from the direct inhibition of fetal hepatic CBG biosynthesis by elevated maternal cortisol exposure and/or limited protein supply during gestation as indicated by a deficiency of essential amino acids in umbilical and fetal blood plasma of identical fetuses [21].

One further critical factor modulating the degree of glucocorticoid access to its intracellular receptor is the presence of glucocorticoid-metabolizing enzymes. Within the brain, 11β-HSD1 is highly expressed in the key loci for glucocorticoid feedback in the HPA axis, notably in the hypothalamus, hippocampus and pituitary, whereas 11β-HSD2 expression is higher in nuclei implicated in mediating central control of the salt/water balance and blood pressure, e.g., the lateral hypothalamus, central nucleus of amygdala and nucleus tractus solitarius [56], [57]. The present results revealed that both inadequate diets affected the expression of 11β-HSD in the brain. There was a higher 11β-HSD1 mRNA expression in the hypothalamus of LP fetuses and a reduced expression of 11β-HSD2 in both LP and HP fetuses. An up-regulation of 11β-HSD1 expression by glucocorticoids has been reported in cell models as well as *in vivo* studies [58], [59]. Stress itself has also been shown to regulate 11β-HSD1 mRNA expression in a time- and tissue-dependent manner. While short-term stressors induce a rise in hippocampal 11β-HSD1 expression, more chronic stress causes a decrease in expression [56]. In a previous study, we demonstrated that a single social isolation caused an increase in cortisol concentrations and 11β-HSD1 mRNA expression in the hypothalamus and hippocampus of postnatal pigs [60]. With respect to the present study, the induction of 11β-HSD1 expression by LP diet would inevitably produce a high intracellular cortisol level that might trigger an intracellular feed-forward mechanism of cortisol formation via the reductase activity of 11β-HSD1 isoenzyme to maximize occupancy and activation of the glucocorticoid receptor. It is assumed that the increase of 11β-HSD1 expression in response to stress or the LP diet may be a compensatory action to increase the negative feedback signal to switch-off the HPA axis [56]. Additionally, fetal exposure to high glucocorticoid levels also may contribute to neurotoxicity [61]. This notion is supported by the present finding that in the fetal hypothalamus, the LP diet during gestation caused a significant reduction of c-fos mRNA expression, a marker of neuronal activity that acts as an inducible transcription factor in a wide range of genes involved in the processes of proliferation, differentiation and cell damage [62]. Repeated stress and chronic glucocorticoid administration have been reported to reduce hypothalamic c-fos expression in rats, indicating that glucocorticoids may be implicated in the regulation of immediate early genes such as c-fos [63]. Moreover, the reduced hypothalamic 11β-HSD2 expression in both LP and HP fetuses may also indicate further glucocorticoid impact. In the brain, 11β-HSD2 is thought to protect the intrinsically non-selective MR from activation by corticosterone and to thus ensure the central regulation of blood pressure and salt balance by aldosterone function [64]. Loss or inhibition of this enzyme results in a syndrome of apparent mineralocorticoid excess, resulting in hypertension and salt/water imbalance [65], [66]. In regards to the present results, we conclude that the maternal LP diet affects the pre-receptor metabolism of both the GR and MR in fetal brain, whereas the maternal HP diet has a negative effect on MR activation. Furthermore, there was also a negative correlation between maternal diurnal cortisol concentrations in LP sows and 11β-HSD1 expression in the fetal hippocampus, which indicates a stronger impact of the LP diet at different glucocorticoid feedback sites.

Taken together, our results demonstrate that in pigs, both the LP and HP diets fed throughout gestation may increase the maternal cortisol concentration and affect the central ‘setting’ of the fetal cortisol regulation. The alterations of hormones and gene expression demonstrate glucocorticoid-dependent effects that were not modulated by placental 11β-HSD2 regulation as reported for other species [50], [67]. However, the specific mechanisms by which unbalanced protein and carbohydrate intake alter the fetal HPA axis involve complex hormonal and neuronal interactions and remain unclear. A previous study by our group with the same experimental design showed that a HP diet during gestation in sows stimulated lipolysis, ureagenesis and gluconeogenesis, reflecting a state of metabolic energy and net glucose deficit, whereas the LP diet caused an absolute deficiency of essential amino acids (as indicated by decreased urea and serum protein levels) and altered lipoprotein metabolism [68], which may point to different pathways involved in glucocorticoid metabolism under the influence of these two diets.

In conclusion, our results demonstrate for the first time that both the low protein∶high carbohydrate diet and the high protein∶low carbohydrate diet fed during gestation in pigs present a metabolic stress to the mother and may have long-lasting consequences for stress coping and health in the offspring. The study highlights the importance of the maternal dietary protein∶carbohydrate ratio during gestation on fetal gene expression involved in central glucocorticoid action and regulatory pathways of adaptation.

## References

[1] Barker DJ, Gluckman PD, Godfrex KM, Harding JE, Owens JA, et al (1993) Fetal nutrition and cardiovascular disease in adult life. Lancet 341: 938–941. 8096277 10.1016/0140-6736(93)91224-a

[2] Kapoor A, Dunn EA, Kostaki A, Andrews MH, Matthews SG (2006) Fetal programming of hypothalamo-pituitary-adrenal (HPA) function: prenatal stress and glucocorticoids. J Physiol 572: 31–44. 16469780 10.1113/jphysiol.2006.105254 PMC1779638

[3] Seckl JR, Holmes MC (2007) Mechanisms of disease: glucocorticoids, their placental metabolism and fetal ‘programming’ of adult pathophysiology. Nat Clin Pract Endocrinol Metab 3: 479–488. 17515892 10.1038/ncpendmet0515

[4] Phillips DI, Barker DJ, Fall CH, Seckl JR, Whorwood CB, et al (1998) Elevated plasma cortisol concentrations: a link between low birth weight and the insulin resistance syndrome? J Clin Endocrinol Metab 83: 747–760. 10.1210/jcem.83.3.4634 9506721

[5] Reynolds RM, Walker BR, Sydall HE, Andrew R, Wood PJ, et al (2001) Altered control of cortisol secretion in adult men with low birth weight and cardiovascular risk factors. J Clin Endocrinol Metab 86: 245–250. 11232008 10.1210/jcem.86.1.7145

[6] Bertram CE, Hanson MA (2002) Prenatal programming of postnatal endocrine responses by glucocorticoids. Reproduction 124: 459–467. 12361463 10.1530/rep.0.1240459

[7] Bertram CE, Trowers AR, Copin N, Jackson AA, Whorwood CB (2001) The maternal diet during pregnancy programs altered expression of the GR and 11β-HSD2: potential molecular mechanism underlying the programming of hypertension in utero. Endocrinology 142: 2841–2853. 11416003 10.1210/endo.142.7.8238

[8] Lesage J, Blondeau B, Grino M, Breant B, Dupouy JP (2001) Maternal undernutrition during late gestation induces fetal overexposure to glucocorticoids and intrauterine growth retardation, and disturbs the hypothalamo-pituitary adrenal axis in the newborn rat. Endocrinology 142: 1692–1702. 11316731 10.1210/endo.142.5.8139

[9] Daenzer M, Ortman S, Klaus S, Metges CC (2002) Prenatal high protein exposure decreases energy expenditure and increases adiposity in young rats. J Nutr 132: 142–144. 11823569 10.1093/jn/132.2.142

[10] Herrick K, Phillips DIW, Haselden S, Shiell AW, Campell-Brown M, et al (2003) Maternal consumption of a high-meat, low-carbonate diet in late pregnancy: relation to adult cortisol concentrations in the offspring. J Clin Endocrinol Metab 88: 3554–3560. 12915635 10.1210/jc.2003-030287

[11] Thone-Reineke C, Kalk P, Dorn M, Klaus S, Simon K, et al (2006) High-protein nutrition during pregnancy and lactation programs blood pressure, food efficiency, and body weight of the offspring in a sex-dependent manner. Am J Physiol Regul Integr Comp Physiol 291: R1025–R1030. 16675628 10.1152/ajpregu.00898.2005

[12] Bamberger CM, Schulte HM, Chrousos GP (1996) Molecular determinants of glucocorticoid receptor function and tissue sensitivity to glucocorticoids. Endocr Rev 17: 245–261. 8771358 10.1210/edrv-17-3-245

[13] Draper N, Stewart PM (2005) 11β-Hydroxysteroid dehydrogenase and the pre-receptor regulation of corticosteroid hormone action. J Endocrinol 186: 251–271. 16079253 10.1677/joe.1.06019

[14] Clarke KA, Ward JW, Forhead AJ, Giussani DA, Fowden AL (2002) Regulation of 11β-hydroxysteroid dehydrogenase type 2 activity in ovine placenta by fetal cortisol. J Endocrinol 172: 527–534. 11874701 10.1677/joe.0.1720527

[15] Seckl JR (2001) Glucocorticoid programming of the fetus; adult phenotypes and molecular mechanisms. Mol Cell Endocrinol 185: 61–71. 11738795 10.1016/s0303-7207(01)00633-5

[16] Klemcke HG, Christenson RK (1996) Porcine placental 11β-hydroxysteroid dehydrogenase activity. Biol Reprod 55: 217–223. 8793078 10.1095/biolreprod55.1.217

[17] Klemcke HG, Sampath Kumar R, Yang K, Vallet JL, Christenson RK (2003) 11β-hydroxysteroid dehydrogenase and glucocorticoid receptor messenger RNA expression in porcine placentae: effects of stage of gestation, breed, and uterine environment. Biol Reprod 69: 1945–1950. 12904312 10.1095/biolreprod.103.018150

[18] Pond WG, Maurer RR, Mersmann HJ, Cummins S (1992) Response of fetal and newborn piglets to maternal protein restriction during early or late pregnancy. Growth Dev Aging 56: 115–127. 1428413

[19] Schoknecht PA, Newton GR, Weise DE, Pond WG (1994) Protein restriction in early pregnancy alters fetal and placental growth and allantoic fluid proteins in swine. Theriogenology 42: 217–226. 16727528 10.1016/0093-691x(94)90265-8

[20] Metges CC, Lang IS, Henning U, Brüssow KP, Kanitz E, et al (2012) Intrauterine growth retarded progeny of pregnant sows fed high protein:low carbohydrate diet is related to metabolic energy deficit. PLoS One 7: e31390. 22328932 10.1371/journal.pone.0031390 PMC3273459

[21] Metzler-Zebeli BU, Lang IS, Görs S, Brüssow KP, Henning U, et al (2012) High-protein–low-carbohydrate diet during pregnancy alters maternal plasma amino acid concentration and placental amino acid extraction but not fetal plasma amino acids in pigs. Br J Nutr doi:10.1017/S0007114512000414. 10.1017/S0007114512000414 22456348

[22] Rehfeld C, Lang IS, Görs S, Henning U, Kalbe C, et al (2011) Limited and excess dietary protein during gestation affects growth and compositional traits in gilts and impairs fetal growth. J Anim Sci 89: 329–34. 20889684 10.2527/jas.2010-2970

[23] Rehfeld C, Lefaucheur L, Block J, Stabenow B, Pfuhl R, et al (2012) Limited and excess protein intake of pregnant gilts differently affects body composition and cellularity of sketal muscle and subcutaneous adipose tissue of newborn and weanling piglets. Eur J Nutr 51: 151–165. 21559991 10.1007/s00394-011-0201-8

[24] Rehfeld C, Stabenow B, Pfuhl R, Block J, Nürnberg G, et al (2012) Effects of limited and excess protein intakes of pregnant gilts on carcass quality and cellular properties of skeletal muscle and subcutaneous adipose tissue in fatting pigs. J Anim Sci 90: 184–196. 21890499 10.2527/jas.2011-4234

[25] Lind NM, Moustgaard A, Jelsing J, Vajta G, Cumming P, et al (2007) The use of pigs in neuroscience: Modeling brain disorders. Neurosci Biobehav Rev 31: 728–751. 17445892 10.1016/j.neubiorev.2007.02.003

[26] Spurlock ME, Gabler NK (2008) The development of porcine models of obesity and metabolic syndrome. J Nutr 138: 397–402. 18203910 10.1093/jn/138.2.397

[27] GfE (Gesellschaft für Ernährungsphysiologie) (2006) Empfehlungen zur Energie- und Nährstoffversorgung von Schweinen (Recommendations of energy and nutrient intake in pigs). Frankfurt am Main: DLG-Verlags GmbH, Frankfurt am Main, Germany.

[28] Felix B, Leger ME, Albe-Fessard D (1999) Stereotaxic atlas of the pig brain. Amsterdam: Elsevier. 10.1016/s0361-9230(99)00012-x 10466025

[29] Kanitz E, Tuchscherer M, Tuchscherer A, Stabenow B, Manteuffel G (2002) Neuroendocrine and immune responses to acute endotoxemia in suckling and weaned piglets. Biol Neonate 81: 203–209. 11937727 10.1159/000051535

[30] Löhrke B, Viergutz T, Krüger B (2005) Polar phospholipids from bovine endogenously oxidized low density lipoprotein interferes with follicular thecal function. J Mol Endocrinol 35: 531–545. 16326838 10.1677/jme.1.01852

[31] Moisan MP, Seckl JR, Edwards CR (1990) 11β-hydroxysteroid dehydrogenase bioactivity and messenger RNA expression in rat forebrain: localization in hypothalamus, hippocampus, and cortex. Endocrinology 127: 1450–1455. 2387261 10.1210/endo-127-3-1450

[32] Lowry OH, Rosebrough NJ, Farr AL, Randall RJ (1951) Protein measurement with Folin reagent. J Biol Chem 193: 265–275. 14907713

[33] SAS Institute Inc (2009) SAS/STAT® 9.2 User's Guide. SAS Institute Inc: Cary, NC.

[34] Pruessner JC, Kirschbaum C, Meinlschmid G, Hellhammer DH (2003) Two formulas for computation of the area under the curve represent measures of total hormone concentration versus time-dependent changes. Psychoneuroendocrinolgy 28: 916–931. 10.1016/s0306-4530(02)00108-7 12892658

[35] Kattesh HG, Baumbach GA, Gillespie BB, Schneider JF, Murai JT (1997) Distribution between protein-bound and free forms of plasma cortisol in the gilt and fetal pig near term. Biol Neonate 72: 192–200. 9303219 10.1159/000244484

[36] McNeil CJ, Nwagwu MO, Finch AM, Page KR, Thain A, et al (2007) Glucocorticoid exposure and tissue gene expression of 11β HSD-1, 11β HSD-2, and glucocorticoid receptor in a porcine model of differential fetal growth. Reproduction 133: 653–661. 17379659 10.1530/rep.1.01198

[37] Galler JR, Ross RN (1993) Malnutrition and mental development. In: Suskind RM, Lewinter-Suskind L , editors. Textbook of pediatric nutrition. New York: Raven Press. pp. 173–179.

[38] Belda X, Ons S, Carrasco J, Armario A (2005) The effects of chronic food restriction on hypothalamic-pituitary-adrenal activity depend on morning versus evening availability of food. Pharmacol Biochem Behav 81: 41–46. 15894062 10.1016/j.pbb.2005.02.009

[39] Gold PW, Gwirtsman H, Avgerinos PC, Nieman LK, Gallucci WT, et al (1986) Abdominal hypothalamic-pituitary-adrenal function in anorexia nervosa. Pathophysiologic mechanism in underweight and weight-corrected patients. N Engl J Med 4: 1335–1342. 10.1056/NEJM198605223142102 3010109

[40] Jacobson L, Zurakowski D, Majzoub JA (1997) Protein malnutrition increases plasma adrenocorticotropin and anterior pituitary proopiomelancortin messenger ribonucleic acid in the rat. Endocrinology 138: 1048–1057. 9048608 10.1210/endo.138.3.5011

[41] Lesage J, Sebaai N, Leonhard M, Dutriez-Casteloot, Breton C, et al (2006) Perinatal maternal undernutrition programs the offspring hypothalamo-pituitary-adrenal (HPA) axis. Stress 9: 183–198. 17175504 10.1080/10253890601056192

[42] Galler JR, Shumsky JS, Morgane PJ (1997) Malnutrition and brain development. In: Walker WA, editor. Nutrition in pediatrics. New York: Plenum Press. pp. 196–212.

[43] Seckl JR, Benediktsson R, Lindsay RS, Brown RW (1995) Placental 11 beta-hydroxysteroid dehydrogenase and the programming of hypertension. J Steroid Biochem Mol Biol 55: 447–455. 8547169 10.1016/0960-0760(95)00193-x

[44] Benediktsson R, Calder AA, Edwards CR, Seckl JR (1997) Placental 11beta-hydroxysteroid dehydrogenase: a key regulator of fetal glucocorticoid exposure. Clin Endocrinol 46: 161–166. 10.1046/j.1365-2265.1997.1230939.x 9135697

[45] Klemcke HG (1995) Placental metabolism of cortisol at mid and late gestation in swine. Biol Reprod 53: 1293–1301. 8562684 10.1095/biolreprod53.6.1293

[46] Klemcke HG (2000) Dehydrogenase and oxoreduktase activities of porcine placental 11β-hydroxysteroid dehydrogenase. Life Sci 66: 1045–1052. 10724451 10.1016/s0024-3205(99)00669-4

[47] Burton PJ, Waddell BJ (1994) 11β-hydroxysteroid dehydrogenase in the rat placenta: developmental changes and the effect of altered glucocorticoid exposure. J Endocrinol 143: 505–513. 7836896 10.1677/joe.0.1430505

[48] Stewart PM, Rogerson FM, Mason JI (1995) Type 2 11β-hydroxysteroid dehydrogenase messenger ribonucleic acid and activity in human placenta and fetal membranes: its relationship to birth weight and putative role in fetal adrenal steroidogenesis. J Clin Endocrinol Metab 80: 885–890. 7883847 10.1210/jcem.80.3.7883847

[49] Langley-Evans SC, Phillips GJ, Benediktsson R, Gardner DS, Edwards CR, et al (1996) Protein intake in pregnancy, placental glucocorticoid metabolism and the programming of hypertension in the rat. Placenta 17: 169–172. 8730887 10.1016/s0143-4004(96)80010-5

[50] Whorwood CB, Firth KM, Budge H, Symonds ME (2001) Maternal undernutrition during early to midgestation programs tissue-specific alterations in the expression of the glucocorticoid receptor, 11β-hydroxysteroid dehydrogenase isoforms, and type 1 angiotensin II receptor in neonatal sheep. Endocrinology 142: 2824–2864. 10.1210/endo.142.7.8264 11416004

[51] Gnanalingham MG, Williams P, Wilson V, Bispham J, Hyatt MA, et al (2007) Nutritional manipulation between early to mid-gestation: effects on uncoupling protein-2, glucocorticoid sensitivity, IGF-I receptor and cell proliferation but not apoptosis in the ovine placenta. Reproduction 134: 615–623. 17890297 10.1530/REP-06-0369

[52] Bright GM (1995) Corticosteroid-binding globulin influences kinetic parameters of plasma cortisol transport and clearance. J Clin Endocrinol Metab 80: 770–775. 7883829 10.1210/jcem.80.3.7883829

[53] Siiteri PK, Murai JT, Hammond GL, Nisker JA, Raymoure WJ, et al (1982) The serum transport of steroid hormones. Recent Prog Horm Res 38: 457–510. 6750727 10.1016/b978-0-12-571138-8.50016-0

[54] Walker CD, Akana SF, Cascio CS, Dallman MF (1990) Adrenalectomy in the neonate: adult-like adrenocortical system responses to both removal and replacement of corticosterone. Endocrinology 127: 823–842. 2164922 10.1210/endo-127-2-832

[55] Heo J, Kattesh HG, Roberts MP, Morrow JL, Dailey JW, et al (2005) Hepatic corticosteroid-binding globulin (CBG) messenger RNA expression and plasma CBG concentrations in young pigs in response to heat and social stress. J Anim Sci 83: 208–215. 15583061 10.2527/2005.831208x

[56] Holmes MC, Seckl JR (2006) The role of 11ß-hydroxysteroid dehydrogenase in the brain. Mol Cell Endocrinol 248: 9–14. 16413106 10.1016/j.mce.2005.12.002

[57] Seckl JR (2004) 11ß-hydroxysteroid dehydrogenases: changing glucocorticoid action. Curr Opin Pharmacol 4: 597–602. 15525550 10.1016/j.coph.2004.09.001

[58] Sun K, He K, Yang K (2002) Intracrine induction of 11β-hydroxysteroid dehydrogenase type 1 expression by glucocorticoid potentiates prostaglandin production in the human chorionic trophoblast. Biol Reprod 67: 1450–1455. 12390875 10.1095/biolreprod.102.005892

[59] Wan S, Hao R, Sun K (2005) Repeated maternal dexamethasone treatments in late gestation increases 11β-hydroxysteroid dehydrogenase type 1 expression in the hippocampus of newborn rat. Neurosci Lett 382: 96–101. 15911129 10.1016/j.neulet.2005.02.066

[60] Kanitz E, Puppe B, Tuchscherer M, Heberer M, Viergutz T, et al (2009) A single exposure to social isolation in domestic piglets activates behavioural arousal, neuroendocrine stress hormones, and stress-related gene expression in the brain. Physiol Behav 98: 176–185. 19460392 10.1016/j.physbeh.2009.05.007

[61] Uno H, Lohmiller L, Thieme C, Kemnitz JW, Engle MJ, et al (1990) Brain damage induced by prenatal exposre to dexamethasone in fetal rhesus macaques. Hippocampus Dev Brain Res 53: 157–167. 2357788 10.1016/0165-3806(90)90002-g

[62] Herdegen T, Leah JD (1998) Inducible and constitutive transcription factors in the mammalian nervous system: control of gene expression by Jun, Fos and Krox, and CREB/ATF proteins. Brain Res Brain Res Rev 28: 370–490. 9858769 10.1016/s0165-0173(98)00018-6

[63] Umemoto S, Kawai Y, Ueyama T, Senba E (1997) Chronic glucocorticoid administration as well as repeated stress affects the subsequent acute immobilization stress-induced expression of immediate early genes but not that of NGFI-A. Neuroscience 80: 763–773. 9276492 10.1016/s0306-4522(97)00050-x

[64] Gomez-Sanchez EP (1986) Intracerebroventricular infusion of aldosterone induces hypertension in rats. Endocrinology 118: 819–823. 3943493 10.1210/endo-118-2-819

[65] Gomez-Sanchez EP, Gomez-Sanchez CE (1992) Central hypertensinogenic effects of glycyrrhizic acid and carbenoxolone. Am J Physiol 263: E1125–E1130. 1476186 10.1152/ajpendo.2006.263.6.E1125

[66] White PC, Mune T, Agarwal AK (1997) 11 beta-hydroxysteroid dehydrogenase and the syndrome of apparent mineralocorticoid excess. Endocr Rev 18: 135–156. 9034789 10.1210/edrv.18.1.0288

[67] Mairesse J, Lesage J, Breton C, Breant B, Hahn T, et al (2007) Maternal stress alters endocrine function of the feto-placental unit in rats. Am J Physiol Endocrinol Metab 292: E1526–E1533. 17264224 10.1152/ajpendo.00574.2006

[68] Stimson RH, Lobley GE, Maraki I, Morton NM, Andrew R, et al (2010) Effects of proportions of dietary macronutrients on glucocorticoid metabolism in diet-induced obesity in rats. PLoS One 5: e8779. 20098742 10.1371/journal.pone.0008779 PMC2808251

